# Positive Association of Ascorbate and Inverse Association of Urate with Cognitive Function in People with Parkinson’s Disease

**DOI:** 10.3390/antiox9100906

**Published:** 2020-09-23

**Authors:** Emma S. Spencer, Toni Pitcher, Gabriel Veron, Tracey Hannam, Michael MacAskill, Tim Anderson, John Dalrymple-Alford, Anitra C. Carr

**Affiliations:** 1 Nutrition in Medicine Research Group, Department of Pathology and Biomedical Science, University of Otago, Christchurch 8011, New Zealand; emma.spencer@otago.ac.nz; 2 Department of Medicine, University of Otago, Christchurch 8011, New Zealand; toni.pitcher@otago.ac.nz (T.P.); michael.macaskill@otago.ac.nz (M.M.); tim.anderson@otago.ac.nz (T.A.); john.dalrymple-alford@canterbury.ac.nz (J.D.-A.); 3 New Zealand Brain Research Institute, Christchurch 8011, New Zealand; 4 Centre for Postgraduate Nursing Studies, University of Otago, Christchurch 8011, New Zealand; verga229@student.otago.ac.nz (G.V.); hantr419@student.otago.ac.nz (T.H.); 5 School of Psychology, Speech, and Hearing, University of Canterbury, Christchurch 8140, New Zealand

**Keywords:** vitamin C, ascorbate, ascorbic acid, Parkinson’s disease, cognitive impairment, MoCA, antioxidant, oxidative stress

## Abstract

Oxidative stress is thought to contribute to the aetiology of neurological disorders such as Parkinson’s disease. Ascorbate (vitamin C) is a potent antioxidant and is associated with neurological and cognitive function. In this study we assessed the ascorbate status of a cohort of people with Parkinson’s disease (*n* = 215), aged 50–90 years, compared with a cohort of age matched healthy controls (*n* = 48). The study sample’s cognitive status ranged from normal to mild cognitive impairment and dementia. There was no difference between the Parkinson’s disease and healthy control groups with respect to mean ascorbate status, however, a higher proportion of participants with Parkinson’s disease had hypovitaminosis C (i.e., <23 μmol/L) compared with healthy controls (20% vs. 8%, respectively). Within the Parkinson’s disease group, Montreal Cognitive Assessment (MoCA) scores correlated positively with ascorbate concentrations, with higher ascorbate status associated with better cognitive function (*r* = 0.14, *p* = 0.045). Participants with hypovitaminosis C had significantly lower MoCA scores relative to participants with ascorbate concentrations >23 µmol/L (*p* = 0.014). Ascorbate concentrations were significantly lower in the cognitively impaired subgroup compared with the normal cognition subgroup in the Parkinson’s disease cohort (*p* = 0.03). In contrast, urate showed an inverse correlation with cognitive function (*r* = −0.19, *p* = 0.007), with higher urate concentrations observed in the cognitively impaired subgroup compared with the normal cognition subgroup (*p* = 0.015). There was an inverse association between ascorbate status and urate concentrations (*r* = −0.15, *p* = 0.017). Plasma protein carbonyls, a measure of systemic oxidative stress, were not significantly different between the Parkinson’s disease cohort and healthy controls, and there was no association with cognitive function (*r* = 0.09, *p* = 0.19) or with ascorbate status (*r* = −0.05, *p* = 0.45). Overall, our study showed ascorbate status was positively associated with cognitive function in Parkinson’s disease, suggesting that longitudinal studies investigating the temporal sequence of cognitive decline and ascorbate status are warranted.

## 1. Introduction

Parkinson’s disease is the most common age-related neurodegenerative disorder after Alzheimer’s disease [1]. More than 10 million people currently live with Parkinson’s disease, and prevalence is expected to double in 20 years [2,3]. Once diagnosed, substantial loss of dopaminergic neurons in the substantia nigra pars compacta and depleted dopamine availability in the striatum is already evident [4]. Diagnosis is based on typical motor symptoms, especially bradykinesia, muscular rigidity, and tremor at rest [5]. No medications yet exist that can slow the progression of Parkinson’s disease [4]. Interventions treat symptoms only, and are primarily for improving health-related quality of life [6].

The quality of life of people with Parkinson’s disease is also impacted by many nonmotor features, both before and after diagnosis. Nonmotor changes include sleep, autonomic, olfactory, pain, and psychiatric symptoms [6]. People with Parkinson’s disease also experience cognitive decline, often the most disabling nonmotor feature [7]. The cognitive decline frequently becomes severe enough to significantly affect every-day cognition, leading to a dementia diagnosis, institutional admission, and reduced lifespan [4,6].

Meta-analysis has indicated that people with Parkinson’s disease dementia tend to have lower serum urate concentrations than controls, and there was a positive correlation between serum urate and cognitive function in people with Parkinsons’ disease dementia [8]. Urate, a metabolite of purine nucleotides, is an antioxidant in blood and brain tissue that exerts neuroprotective effects by scavenging reactive oxygen species [9,10]. Although urate has been associated with cognitive function in older people, the data is somewhat conflicting and may depend on the aetiology of the dementia, with protective effects being observed in Parkinson’s dementia and Alzheimer’s disease, but not in vascular dementia [11].

Plasma ascorbate concentrations have also been associated with cognition in the general population, with cognitively impaired groups having lower mean ascorbate status than cognitively intact groups [12]. Ascorbate (vitamin C) is a potent antioxidant obtained through the diet. In the brain, ascorbate is maintained at high concentrations relative to other organs [13], and low levels are linked to neurodegenerative diseases [14,15]. Previous research has indicated that patients with Parkinson’s disease have significantly lower concentrations of plasma ascorbate and higher concentrations of oxidative stress markers compared to healthy controls [16,17]. Furthermore, decreased concentrations of leukocyte ascorbate is correlated with disease severity assessed by Hoehn–Yahr stage in Parkinsons’ disease patients [18].

The current study examined the association between plasma ascorbate and cognitive function in participants with Parkinson’s disease and in healthy controls using the Montreal Cognitive Assessment (MoCA). To capture the spectrum of cognition in Parkinson’s disease, we included patients who had been classified as having either normal cognition (PD-N), mild cognitive impairment (PD-MCI), or dementia (PD-D). Ascorbate has numerous biosynthetic and regulatory functions in addition to its well-known antioxidant activities, so we assessed both antioxidants and oxidatively damaged molecules. For the latter, we examined protein carbonyls, as a stable marker of systemic oxidative stress.

## 2. Materials and Methods

### 2.1. Parameters of the Study Sample

A cohort of people with Parkinson’s disease (*n* = 215) was recruited by the New Zealand Brain Research Institute (NZBRI) and blood samples were collected between August 2016 and February 2019. They were diagnosed using the Movement Disorder Society Clinical Diagnostic Criteria for Parkinson’s disease (MSD-PD) [19]. For comparison, blood samples were collected from a cohort of healthy individuals (*n* = 48) matched for age and gender.

### 2.2. Neuropsychological Testing

Comprehensive assessment meeting the Movement Disorders Society (MDS) Task Force level II criteria classified Parkinson’s disease patients as either PD-N, PD-MCI, or PD-D using neuropsychological test spanning five cognitive domains (executive function; attention, working memory and processing speed; learning and memory; visuospatial/visuoperceptual function; language) [20,21]. PD-MCI cases did not have significantly impaired functional activities of daily living and scored ≥1.5 SD below normative data on two tests in at least one of the five cognitive domains [22]. PD-D cases had poor cognitive performance (i.e., 2 SD below normative data in at least two of the five cognitive domains) associated with significant impairment in everyday functional activities, not attributed to motor impairments [20].

### 2.3. Montreal Cognitive Assessment (MoCA)

The majority of participants (*n* = 260) from both the Parkinson’s disease and control groups were evaluated with the MoCA, a global mental status test [23,24]. MoCA scores range from 0 to 30. MoCA scores in PD correspond to cognitive status as determined by a full neuropsychological evaluation (26–30: normal cognitive status, 21–25: mild cognitive impairment, <21: dementia).

### 2.4. Hoehn and Yahr Scale

Most of the Parkinson’s disease cohort (*n* = 187) were evaluated with the modified Hoehn and Yahr scale at the time of giving their blood sample [25]. This evaluation stages motor symptoms on an eight-point scale from stage 0, no signs of disease, to stage 5, individual is wheelchair bound or bedridden unless assisted.

### 2.5. Unified Parkinson’s Disease Rating Scale (UPDRS)

Motor functions were also assessed in the majority of the Parkinson’s disease group (*n* = 187) using part III of the Movement Disorder Society revision of the Unified Parkinson’s Disease Rating Scale (MDS-UPDRS) [26]. This instrument uses 18 items, rating symptoms such as tremor, speech, facial expression, gait, and rigidity on a five-point scale from 0 (normal function) to 4 (severe dysfunction). In Part III, scores >32 indicate moderate symptom severity and >58 indicates severe symptom severity (maximum score = 132).

### 2.6. Blood Sample Collection and Processing

Nonfasting blood samples were collected by venipuncture into lithium heparin anticoagulant vacutainers and placed immediately on ice for biomarker stability [27]. Samples were centrifuged at 4000× *g* for 10 min at 4 °C, and the separated plasma was stored at −80 °C.

### 2.7. Ascorbate and Urate Measurement by HPLC

The total ascorbate and urate content of plasma was analysed using reverse-phase high-performance liquid chromatography (HPLC) with electrochemical detection, as previously described [28]. Prior to HPLC analysis, the samples were treated with an equal volume of ice-cold 0.54 M perchloric acid (PCA) solution containing 100 μmol/L of the metal chelator diethylenetriaminepentaacetic acid (DTPA) to precipitate proteins and stabilise the ascorbate. To recover any ascorbate that had become oxidised during processing and/or storage, the PCA-supernatant was treated with 10% *v/v* tris(2carboxyethyl)phosphine (TCEP; 350 mmol/L) for 3 h at 4 °C. Samples were separated on a Synergi 4 µ Hydro-RP 80A 150 × 4.6 mm column (Phenomenex NZ Ltd., Auckland, New Zealand) using a Dionex Ultimate 3000 HPLC unit with a refrigerated autosampler and an ESA Coulochem II electrochemical detector at +200 mV electrode potential and 20 µA sensitivity (Thermo Fisher Scientific, Auckland, New Zealand). The mobile phase comprised 80 mM sodium acetate buffer, pH 4.8, containing DTPA (0.54 mmol/L) and freshly added paired-ion reagent n-octylamine (1 μmol/L), delivered at a flow rate of 1.2 mL/min. A standard curve of sodium-L-ascorbate (1.25–20 μmol/L) and uric acid (1.25–20 μmol/L), standardised spectrophotometrically, were freshly prepared for each HPLC run in 77 mmol/L perchloric acid containing 100 µmol/L DTPA.

### 2.8. Protein Carbonyl Measurement by ELISA

Plasma protein carbonyl concentrations were measured using an enzyme-linked immunosorbent assay (ELISA) as previously described [29]. Samples and standards were derivatised with 0.1 mM dinitrophenylhydrazine (DNP) in 6 M guanidine hydrochloride for 45 min while at room temperature. Each standard and sample was then diluted in PBS, and 200 µL aliquots were added into the ELISA plate wells in triplicate and incubated at 4 °C overnight. The ELISA plate was washed three times with PBS and blocked with PBS containing 0.1% (*v*/*v*) Tween 20 at room temperature for 30 min. The plate was then washed again and the bound protein was probed with a biotinylated anti-DNP antibody at 1:1000 in PBS containing 0.1% (*v*/*v*) Tween 20 for 1 h at 37 °C. The plate was rewashed, and probed with streptavidin-linked horseradish peroxidase at a 1:1000 dilution in PBS containing 0.1% (*v*/*v*) Tween 20 and incubated for 1 h at room temperature. The plate was washed again and Chromatin reagent added to each well. The colour development process was stopped after approximately 5 min by adding 2.5 M sulfuric acid. Absorbance was measured at 450 nm on a Thermo Scientific Multiskan GO plate reader using Thermo Scientific SkanIt 5.0 software (Thermo Fisher Scientific, Auckland, New Zealand). Protein carbonyl concentrations were calculated using a standard curve plotted using the pmol/mg protein carbonyl concentration of the standards and their absorbance values.

### 2.9. Statistical Analyses

Pearson’s linear correlation analyses were used to express the relationship between continuous variables and unpaired *t*-tests were used to compare groups. Categorical data were examined using Chi-squared tests with 2 × 2 contingency tables. Statistical analyses were conducted using Microsoft Excel data analysis add-in (Microsoft, Auckland, NZ) and GraphPad Prism version 8.0 (GraphPad Software, San Diego, CA, USA). Statistical significance was set at *p* < 0.05.

## 3. Results

### 3.1. Participant Characteristics

In the Parkinson’s disease group (*n* = 215), 70% were male and the mean age was 73 ± 7 years (with a range of 51–89 years; Table 1). Most participants self-identified as NZ European (*n* = 204, 95%). There were 95 (44%) PD-N, 84 (39%) PD-MCI, and 35 (16%) PD-D; all healthy controls had normal cognition for their age. Mean MoCA scores in the Parkinson’s disease group were significantly lower than those of the healthy controls (24 ± 5 vs. 27 ± 3, respectively, *p* < 0.0001). Hoehn and Yahr stage of 2.6 ± 0.6 indicated mild bilateral involvement on the pull test and was comparable across the PD subgroups. The mean UPDRS part III score of 40 ± 14 indicated that most had moderate Parkinson’s disease motor symptoms when on antiparkinsonian medication.

### 3.2. Ascorbate Status of the Study Population

**Figure 1:**
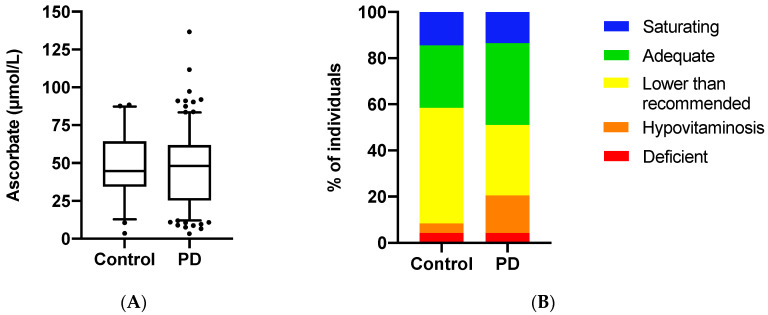
Ascorbate status of the study population. (**A**) Box plots show medians in plasma ascorbate concentrations in the Parkinson’s disease (PD) group (*n* = 214) and control group (*n* = 48). Boundaries are set at the 25th and 75th percentiles, whiskers at 5th and 95th percentiles, outliers are indicated by symbols. Mean plasma ascorbate concentrations were not significantly different between the two groups (*p* = 0.74). (**B**) Proportion of participants in different ascorbate categories. Individuals within the Parkinson’s disease (PD) and control groups were classified as having saturating (>70 μmol/L), adequate (>50 μmol/L), lower than recommended (<50 μmol/L), hypovitaminosis C (<23 μmol/L), and deficient (<11 μmol/L) plasma ascorbate concentrations [30]. Categories are shown as percentages. The PD group had a significantly higher proportion of hypovitaminosis C (*p* = 0.048).

The mean plasma ascorbate concentration for the total cohort was 47 ± 22 μmol/L (*n* = 262). Females had nonsignificantly higher levels than males (50 ± 23 µmol/L vs. 45 ± 22 µmol/L; *p* = 0.07). There was no difference in mean levels between the Parkinson’s disease and control groups (47 ± 23 μmol/L vs. 48 ± 20 μmol/L; *p* = 0.74; Figure 1A). Participants were assigned to five categories based on their ascorbate status: saturating (>70 μmol/L), adequate (>50 μmol/L), lower than recommended (<50 μmol/L), hypovitaminosis C (<23 μmol/L), and deficient (<11 μmol/L) [30]. There was a significantly higher proportion of hypovitaminosis C in the Parkinson’s disease group relative to the healthy controls (20% vs. 8%, *p* = 0.048; Figure 1B).

### 3.3. Ascorbate Status Relative to Cognitive Function and Clinical Parameters

**Figure 2:**
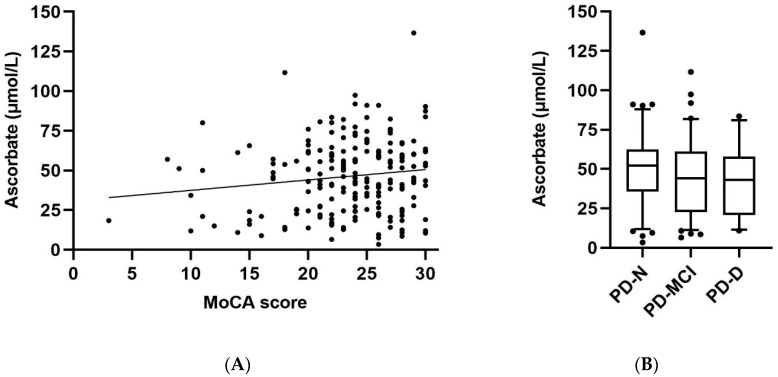
Plasma ascorbate concentrations relative to cognitive function. (**A**) MoCA (Montreal Cognitive Assessment) scores in the Parkinson’s disease group (*n* = 212) showed a significant positive correlation with ascorbate status (*r* = 0.14, *p* = 0.045). (**B**) Plasma ascorbate concentrations in individuals with Parkinson’s disease as per cognitive status subgroups: normal (PD-N, *n* = 95), mild cognitive impairment (PD-MCI, *n* = 84), and dementia (PD-D, *n* = 34); the cognitively impaired group (PD-MCI and PD-D combined, *n* = 118) was significantly lower than the PD-N group (*p* = 0.03). Box plots show medians with boundaries set at 25th and 75th percentiles, whiskers at 5th and 95th percentiles, outliers are indicated by symbols.

In the Parkinson’s disease group (*n* = 212), MoCA scores showed a weak positive correlation with plasma ascorbate concentrations (*r* = 0.14, *p* = 0.045; Figure 2A). Thus, a higher ascorbate status was associated with a better cognitive score. A comparable correlation was observed with the healthy controls (*r* = 0.29, *p* = 0.048). When the MoCA data was analysed relative to ascorbate status, participants with hypovitaminosis C (i.e., <23 µmol/L) had significantly lower MoCA scores relative to participants with ascorbate concentrations >23 µmol/L (21.9 ± 6.0 vs. 23.9 ± 4.4, *p* = 0.014). The mean ascorbate concentration in the cognitively impaired group (PD-MCI and PD-D combined; 43 ± 23 μmol/L, *n* = 118) was significantly lower than that of the PD-N group (50 ± 23 μmol/L, *n* = 95, *p* = 0.03; Figure 2B). There was no evidence of a correlation between ascorbate status and the Hoehn and Yahr scale (*r* = −0.03, *p* = 0.64), or between ascorbate status and UPDRS Part III scores (*r* = −0.06, *p* = 0.42) in the Parkinson’s disease group.

### 3.4. Urate Concentrations Relative to Cognitive Function and Clinical Parameters

**Figure 3:**
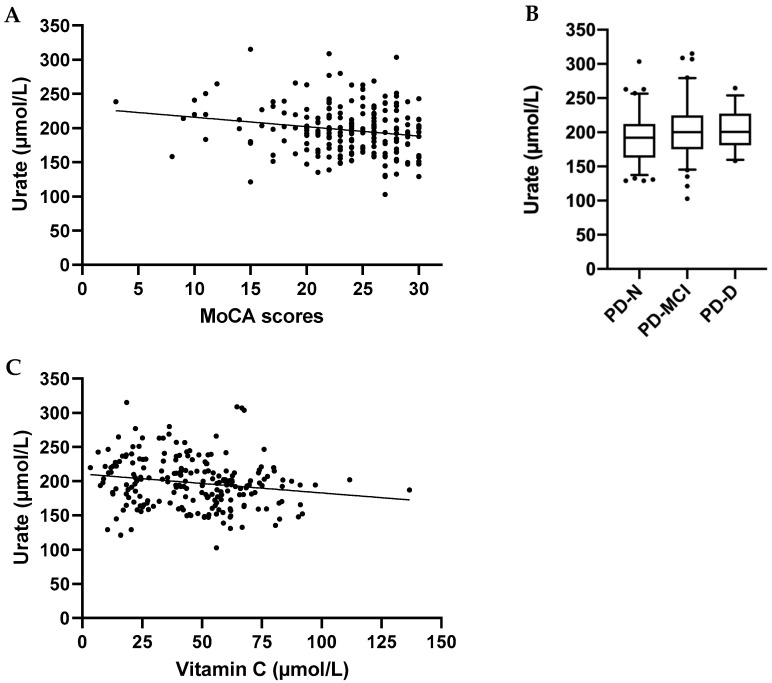
Plasma urate concentrations relative to cognitive function and plasma ascorbate status. (**A**) Montreal Cognitive Assessment (MoCA) scores in the Parkinson’s disease group (*n* = 212) showed a significant inverse correlation with urate status (*r* = −0.19, *p* = 0.007). (**B**) Plasma urate concentrations in individuals with Parkinson’s disease as per cognitive status subgroups: normal (PD-N, *n* = 95), mild cognitive impairment (PD-MCI, *n* = 84), and dementia (PD-D, *n* = 34). The cognitively impaired group (PD-MCI and PD-D combined, *n* = 118) was significantly higher than the PD-N group (*p* = 0.015). Box plots show medians with boundaries set at 25th and 75th percentiles, whiskers at 5th and 95th percentiles, outliers are indicated by symbols. (**C**) Plasma urate concentrations in the Parkinson’s disease group (*n* = 212) show a weak inverse correlation with plasma ascorbate concentrations (*r* = −0.15, *p* = 0.017).

Plasma urate concentrations were not significantly different between the Parkinson’s disease and control groups (198 ± 36 μmol/L vs. 207 ± 31 μmol/L, respectively, *p* = 0.08). None of the participants had hyperuricemia, defined as plasma concentrations >360 µmol/L. MoCA scores analysed against plasma urate concentrations in the Parkinson’s disease group (*n* = 212) showed a small inverse association (*r* = −0.19, *p* = 0.007; Figure 3A), i.e., higher urate was associated with worse cognitive scores. In contrast, no correlation was observed between MoCA scores and urate concentrations in the healthy cohort (*r* = 0.11, *p* = 0.4). The urate values in the cognitively impaired group (PD-MCI and PD-D combined; 203 ± 37 μmol/L, *n* = 118) were significantly higher than the PD-N group (191 ± 35 μmol/L, *n* = 95, *p* = 0.015; Figure 3B). In addition, there was a small but positive correlation between UPDRS part III scores and plasma urate concentrations (*r* = 0.18, *p* = 0.02), suggesting that higher plasma urate is associated with worse motor skill scores. The correlation between plasma urate concentrations and Hoehn and Yahr scores did not reach significance (*r* = 0.14, *p* = 0.06). There was a weak inverse correlation between ascorbate and urate concentrations in the Parkinson’s disease group (*r* = −0.15, *p* = 0.017), with higher plasma ascorbate concentrations associated with lower plasma urate concentrations (Figure 3C).

### 3.5. Protein Carbonyl Concentrations Relative to Cognitive Function and Clinical Parameters

**Figure 4:**
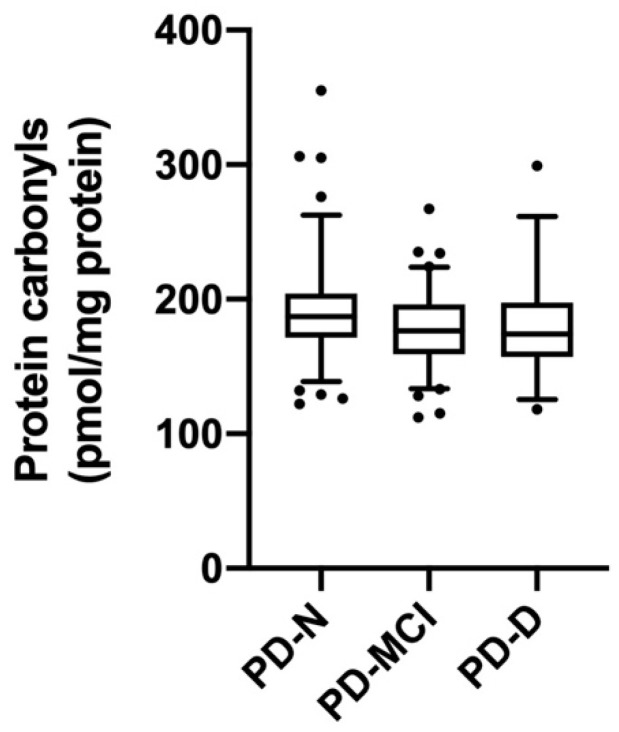
Plasma protein carbonyl concentrations relative to cognitive function. Plasma protein carbonyl concentrations in individuals with Parkinson’s disease as per cognitive status subgroups: normal (PD-N, *n* = 94), mild cognitive impairment (PD-MCI, *n* = 84), and dementia (PD-D, *n* = 34). The cognitively impaired group (PD-MCI and PD-D combined, *n* = 118) was significantly lower than the PD-N group (*p* = 0.007). Box plots show medians with boundaries set at 25th and 75th percentiles, whiskers at 5th and 95th percentiles, outliers are indicated by symbols (two outliers in the PD-N subgroup are not shown on the figure; 969 and 603 pmol/mg protein).

There was no significant difference in protein carbonyl concentrations between the Parkinson’s disease and control groups (190 ± 7 vs. 191 ± 39 pmol/mg protein; *p* = 0.8). Two participants in the Parkinson’s disease group had protein carbonyl concentrations of 969 and 603 pmol/mg protein. The reason for these high concentrations is not known, but did not appear to relate to cognitive function as both were in the PD-N subgroup. Even with exclusion of these two outliers, the protein carbonyl values in the PD-N group (191 ± 36 pmol/mg protein, *n* = 96) were significantly higher than the cognitively impaired group (PD-MCI and PD-D combined; 179 ± 30 pmol/mg protein, *n* = 118; *p* = 0.007; Figure 4). However, MoCA scores analysed against plasma protein carbonyl concentrations in the Parkinson’s disease group (*n* = 212) showed no evidence of association (*r* = 0.09, *p* = 0.16). This was true also for the healthy control group (*r* = 0.07, *p* = 0.65). Additionally, there was no significant correlation between plasma protein carbonyl concentrations and Hoehn and Yahr scores (*r* = −0.12, *p* = 0.09) or UPDRS part III scores (*r* = −0.13, *p* = 0.06) in the Parkinson’s disease group. There was no significant correlation between protein carbonyl concentrations and plasma ascorbate status (*r* = −0.05, *p* = 0.45) or urate concentrations (*r* = −0.06, *p* = 0.4).

## 4. Discussion

In this study, we observed that people with Parkinson’s disease had similar mean plasma ascorbate status to healthy age-matched controls, values which were comparable to a previously reported study in Spain [31]. Although other studies (in India and Brazil) have shown lower ascorbate concentrations in people with Parkinson’s disease compared to healthy controls [16,17], based on the low control values and the less than ideal analytical methodology used, it is possible that these low values were due to artifactual ex vivo oxidation [27]. Alternatively, individuals in low-middle income countries tend to have lower vitamin C status, and thus may be more prone to developing hypovitaminosis C during various disease states [32,33]. Of note, the Parkinson’s disease group in our study had a higher prevalence of hypovitaminosis C, where the subclinical symptoms of vitamin C deficiency start to become apparent [34].

We observed lower ascorbate status associated with cognitive decline in the Parkinson’s disease group, in line with a previous report which indicated decreasing vitamin C status with severity of the disease [18]. Participants with hypovitaminosis C, in particular, had significantly lower mean MoCA scores than those with ascorbate status >23 µmol/L. Previous studies have reported lower ascorbate status in people with cognitive impairment, e.g., Alzheimer’s disease [12]. There are, however, a paucity of studies examining the role of ascorbate in the cognitive decline related to Parkinson’s disease progression. We also observed a positive association of ascorbate status with cognitive function in the healthy control group, which has been observed previously in middle-aged people in the general population [35]. Therefore, the association of ascorbate with cognitive function is not specific to people with neurological disease, but is a more generalisable phenomenon.

There are a number of activities of vitamin C that are believed to contribute to brain function [36]. For example, ascorbate acts as an antioxidant in the brain, which, due to its high metabolic rate, is particularly exposed to oxidative stress. Ascorbate also plays a role in the synthesis of dopamine [14,37]. Additionally, ascorbate has been shown to have a role in the epigenetic mechanisms of gene expression [38]. Abnormalities in the concentrations of the epigenetic marks 5-methyl cytosine and 5-hydroxymethyl cytosine in the brain have been associated with the development of neurodegenerative diseases, including Parkinson’s disease [39,40]. More recently, ascorbate treatment of human induced pluripotent stem cells was shown to elevate gene expressions of the epigenetic enzymes TET2, TET3, and ascorbate transporters, and promote neuronal maturation, synaptic activity, and dopamine release [41].

In our study, urate concentrations were nonsignificantly lower in the Parkinson’s disease group compared with the control group. However, we did observe a small inverse correlation between urate concentrations and MoCA scores in the Parkinson’s disease group, with higher plasma urate concentrations associated with worse cognitive scores. This is consistent with longitudinal studies that reported elevated baseline serum urate concentrations were associated with faster cognitive decline over time [42,43]. There are, however, conflicting reports in the literature around the association of urate with cognitive function. A 2016 systematic review of ≈16,000 participants over 46 papers found no clear association between serum urate levels and cognition [44], although the studies analysed included patients with Alzheimer’s disease, Parkinson’s disease, MCI, and dementia-free people, making interpretation of the results challenging [45]. Additionally, findings may be complicated by the presence of comorbidities such as kidney and cardiovascular disease [46,47].

We observed a weak inverse correlation between plasma ascorbate and urate concentrations, with higher ascorbate associated with lower urate concentrations, as has been observed in other studies [48,49]. The uricosuric effects of ascorbate are thought to be responsible for the reduction in urate levels [50], but it is also thought that ascorbate may inhibit uric acid synthesis [51]. Alternatively, higher ascorbate may indicate a healthier diet, i.e., one with more fresh fruit and vegetables [52], versus a purine-rich diet that can contribute to higher urate concentrations.

As ascorbate and urate had opposite associations with cognitive function, despite both being considered antioxidants, this indicates that other non-antioxidant functions of ascorbate may be playing a role in cognitive function. The lack of an association between MoCA scores and protein carbonyls, a stable marker of oxidative stress, and between protein carbonyls and ascorbate in our study lends confirmation to this premise. Protein carbonyls are a general marker of oxidative stress as they can be formed by a variety of reactive oxygen species and via a number of different reaction pathways [53]. Although protein carbonyls have been negatively associated with MoCA scores in patients with Alzheimer’s disease [54], relatively few studies have measured protein oxidation in people with Parkinson’s disease and these have yielded contradictory results [55,56]. Although elevated lipid oxidation markers have been observed in Parkinson’s disease [16,17], there is as yet no clear link between oxidative stress and cognitive function in Parkinson’s disease. However, assessing peripheral biomarkers of oxidative stress may not accurately reflect processes occurring in the brain as Parkinson’s neuropathology is restricted to a relatively small volume of the brain [57].

A limitation of this observational study is the lack of certainty around direction of causality. Decreased ascorbate levels may lead to worse cognitive status, however, the opposite is also a possibility in that people with reduced cognitive capacity do not eat as well, which can result in nutritional deficiencies. Additionally, plasma ascorbate may simply be a marker of a healthy diet [52], without any direct activity itself on cognitive function. Nevertheless, the vitamin does have biological plausibility with a number of activities that conceivably contribute to brain function [36]. Another potential limitation of the study is the nonfasting nature of the collected blood samples. However, the vitamin C status of people in the hypovitaminosis range is less susceptible to fluctuations as a result of recent dietary intake [58]. Compared with plasma, leukocyte ascorbate concentrations are considered a more stable measure, less affected by transient dietary changes [59]. Interestingly, lymphocyte ascorbate concentrations have been reported to be lower in patients with severe Parkinson’s disease, when compared to patients at less severe stages, suggesting that lymphocyte ascorbate concentrations could be a potential marker for progression of the disease [18].

## 5. Conclusions

Our study showed a positive association of ascorbate status with cognitive function in people with Parkinson’s disease. Due to the uncertainty around the direction of causality, it would be worthwhile to conduct a longitudinal study to probe the temporal sequence of cognitive decline and ascorbate status. This would indicate if further studies investigating the role of ascorbate supplementation in cognitive decline in individuals with Parkinson’s disease are warranted.

## Figures and Tables

**Table 1:** Participant characteristics.

Parameters	Control Group (*n* = 48)	PD Group (*n* = 215)	PD-N Subgroup (*n* = 95)	PD-MCI Subgroup (*n* = 84)	PD-D Subgroup (*n* = 35)
Age, years ^1^	72 ± 7	73 ± 7	70 ± 7	72 ± 7	76 ± 5
Male, *n* (%)	29 (60)	150 (70)	59 (62)	62 (74)	29 (83)
Cognitive status, *n* (%) ^2^					
Normal	45 (94)	95 (44)	-	-	-
MCI	2 (4)	84 (39)	-	-	-
Dementia	1 (2)	35 (16)	-	-	-
MoCA score	27 ± 3	24 ± 5	27 ± 2	23 ± 3	17 ± 5
Hoehn and Yahr scale	-	2.6 ± 0.6	2.4 ± 0.5	2.7 ± 0.7	3.0 ± 0.7
UPDRS part III score	-	40 ± 14	34 ± 13	45 ± 13	49 ± 13

^1^ Data are presented as mean ± SD, unless otherwise specified. ^2^ Cognitive status was not determined for one participant in the PD group. PD, Parkinson’s disease; N, normal; MCI, mild cognitive impairment; D, dementia; MoCA, Montreal Cognitive Assessment; UPDRS, Unified Parkinson’s Disease Rating Score.
